# Laboratory Chamber Evaluation of Flow Air Quality Sensor PM_2.5_ and PM_10_ Measurements

**DOI:** 10.3390/ijerph19127340

**Published:** 2022-06-15

**Authors:** Natalie Crnosija, Misti Levy Zamora, Ana M. Rule, Devon Payne-Sturges

**Affiliations:** 1 Maryland Institute for Applied Environmental Health, University of Maryland School of Public Health, 255 Valley Drive, College Park, MD 20742, USA; dps1@umd.edu; 2 University of Connecticut Health Center, Department of Public Health Sciences, UConn School of Medicine, 263 Farmington Avenue, Farmington, CT 06032-1941, USA; mzamora@uchc.edu; 3 Bloomberg School of Public Health, Environmental Health and Engineering, Johns Hopkins University, 615 N Wolfe St, Baltimore, MD 21205-2103, USA; arule1@jhu.edu

**Keywords:** air pollution, low-cost sensors, particulate matter

## Abstract

The emergence of low-cost air quality sensors as viable tools for the monitoring of air quality at population and individual levels necessitates the evaluation of these instruments. The Flow air quality tracker, a product of Plume Labs, is one such sensor. To evaluate these sensors, we assessed 34 of them in a controlled laboratory setting by exposing them to PM_10_ and PM_2.5_ and compared the response with Plantower A003 measurements. The overall coefficient of determination (R^2^) of measured PM_2.5_ was 0.76 and of PM_10_ it was 0.73, but the Flows’ accuracy improved after each introduction of incense. Overall, these findings suggest that the Flow can be a useful air quality monitoring tool in air pollution areas with higher concentrations, when incorporated into other monitoring frameworks and when used in aggregate. The broader environmental implications of this work are that it is possible for individuals and groups to monitor their individual exposure to particulate matter pollution.

## 1. Introduction

Once the exclusive purview of governmental organizations, air quality monitoring is becoming increasingly individualized and commercialized through the creation and marketing of personal air quality sensors. These sensors can measure a number of ambient air pollutants, including those monitored and regulated by the Environmental Protection Agency (EPA) [1]. The EPA’s stationary monitors are strategically sited, based on factors including the location of population centers and pollution point sources to assess compliance with the National Ambient Air Quality Standards (NAAQS), which include carbon monoxide (CO), lead (Pb), nitrogen dioxide (NO_2_), ozone (O_3_), sulfur dioxide (SO_2_), and particulate matter (PM_10_ and PM_2.5_) [2]. This environmental pollution evaluation paradigm may be shifting because of the ability to collect data without relying on a network of stationary monitors. Novel, mobile sensors allow us to measure an individual’s mobile breathing microenvironment [3], enabled by relatively portable, low-cost sensors.

To evaluate the utility of low-cost sensors, validation procedures, such as those used by Jovašević-Stojanovic et al. (2015), are needed, and have been executed by a number of other researchers [4]. The authors compared two particulate matter sensors, a high-quality scientific monitor (GRIMM Model 1.108 monitor) and a low-cost monitor (Dylos 1700 (Dylos Corporation, Riverside, CA, USA), by testing both monitors in an indoor environment and an outdoor environment and found strong agreement between the two monitors. Lowther et al. (2019) reviewed particulate matter sensor types (optical vs. electric vs. gravimetric) and evaluated the different PM assessment techniques associated with these sensor types [5]. The majority of these sensors measured particle mass rather than particle number, particle size distribution, or particle surface area—the standardization of measurement approaches is a matter that still needs to be addressed by the air quality measurement community, especially with regard to indoor air quality. Regarding the practical utility of these low-cost of sensors, Lowther et al. (2019) state that these types of sensors may be used in city environments, where the internet infrastructure exists to support their use. In their evaluation of five different low-cost optical air quality sensors relative to lab-based instruments, Bulot et al. (2020) were able to identify events of pollutant introduction in laboratory conditions [6]. The sensitivity of these sensors depended on the model of the sensor, demonstrating the variation that different models of similar devices can introduce. The portability of low-cost sensors makes it possible to evaluate spatially variable indoor air quality [5], which can represent the majority of one’s daily ambient air environment. Studies have found that time spent indoors can range between 65% [7] and 91% [8] of subjects’ time each day.

Though these evaluations provide encouraging findings for the development and deployment of low-cost sensors, Castell et al. (2017) asserted that low-cost sensor data may have use in aggregate, but are not directly comparable to those sensors used towards regulatory ends, as these low-cost sensors lack accuracy on an individual level [9]. Given the potential for low-accuracy monitoring by low-cost sensors, Kumar et al. (2015) asked whether collecting data using these tools is worthwhile [10]—as sensors continue to advance in their accuracy and precision, this concern may be increasingly lessened. 

The ability to conduct low-cost air quality monitoring may be particularly valuable for socially and economically vulnerable populations in the United States, who may be differentially affected by under-investigated air-quality issues [11], leading to environmental injustice. As per the Environmental Protection Agency, Environmental Justice is “the fair treatment and meaningful involvement of all people, regardless of race, color, national origin, or income with respect to the development, implementation, and enforcement of environmental laws, regulations, and policies” [12]. The capability of portable sensors to assess pollution locally may be why these sensors have been embraced in environmental justice research, a capacity upon which proponents of these sensors’ validation have remarked [4,9,10]. Hall et al. (2014) suggest that low-cost sensors can play a role in monitoring air-quality holistically through their capacity to increase monitoring’s spatial coverage and potential for improving pollution characterization [13]. Plume Labs’ Flow device offers the ability to measure air quality wherever one goes with the sensor, making it particularly useful for environmental justice projects given its low cost, small size, and attractive design [14].

One organization that conducts validation evaluations of low-cost sensors is the South Coast Air Quality Management District (AQMD), a state agency that monitors the air quality in Southern California and makes recommendations for air pollution control [15]. South Coast AQMD has evaluated the validity and reliability of a number of these types of sensors, including Air Quality Egg, Purple Air, and AeroQual [15]. Plume Lab’s Flow2 low-cost sensor was recently evaluated by South Coast AQMD. They found preliminarily that there was an R^2^ for PM_2.5_ measurement between 0.02 and 0.15 between three Flow 2 units and a Federal Equivalent Monitor T640 (FEM T640) over the course of an hour of laboratory monitoring (Using the Federal Equivalent Monitor GRIMM (FEM GRIMM) PM_2.5_ values, the R^2^ for PM_2.5_ measurement between 0.02 and 0.22 among three Flow 2 units) [16]. Correlation using the Flow 2s and FEM T640 was poorer when the 5 min observation periods were conducted (0.01 < R^2^ < 0.09) and stronger using 24 h of observation R^2^ (0.02 < R^2^ < 0.72). This suggests that the longer the duration, the stronger the association between Flow 2s and FEMs—this finding may be attributed to the design of the Flow, which auto-calibrates through algorithm processes executed through the device’s firmware [17]. Through this process, the more air quality environments to which the device is exposed, the better the sensor’s measurement of pollutants. South Coast AQMD’s analysis featured data from three Flow 2 sensors [16], an approach that many other evaluation studies have used; yet, we do not know whether increasing the number of sensors and aggregating their measurements would produce similar evidence of the correlation between low-cost air quality sensors and high-precision sensors, by virtue of an increased number of personal, mobile sensors that may be required for air-quality monitoring field campaigns.

The purpose of this paper is to present our findings comparing the average PM_2.5_ and PM_10_ measurements of 34 Flow devices to the PM_10_ and PM_2.5_ concentrations simultaneously measured with a previously calibrated and validated sensor (Plantower A003) inside a chamber [18,19].

## 2. Materials and Methods

### 2.1. Low-Cost Sensors Evaluated

Low-cost (~150 USD/sensor) and commercially available to the public, Flow devices measure ambient air quality. Air pollutants (NO_2_, VOCs, PM_10_, and PM_2.5_) are measured by drawing ambient air into the device by a small, internal electric fan through the holes in the body of the device [20]. Once the air sample enters the device, a heated membrane measures the amount of energy needed to maintain the temperature required by the membrane to disintegrate NO_2_ and VOCs. Particulate matter is measured as the amount of laser-produced light that is diffracted when the laser collides with airborne fine particulate taken into the device [21]. Both approaches quantify the concentrations of these pollutants. A Flow device and a smartphone or tablet must be paired through a Bluetooth connection. For the device to properly collect time-stamped sensor air-quality and spatial data, Wi-Fi/mobile data and GPS connectivity with a companion device are also required. The manufacturer recommends that the sensors be exposed to a variety of indoor and outdoor air environments of high- and low-air-pollutant concentration exposures to allow for autocalibration over the course of a week [17]. The manufacturer did not provide a range of measurement. We did not zero the sensor before use, as it would undo this calibration process and it is not expected of the typical user. Once the sensor is fully charged, which takes 2.5 h, the Flow’s charge will last 24 h [22]. 

A previously validated and calibrated sensor [18] (Plantower A003) was used as an instrument to which the Flows could be compared. The Plantower A003 also uses a fan to pull air into a chamber where laser-produced light is diffracted by airborne particulate matter. Those collisions that occur at a 90° angle are then able to be measured by a photodiode detector [23,24]. Plantower A003 has demonstrated high precision (PM_2.5_ measurement precision error = 7% and PM_10_ measurement precision error = 9%) and overall PM_2.5_ accuracy (87%) in the measurement of incense [18].

### 2.2. Chamber Experiments

**Figure 1:**
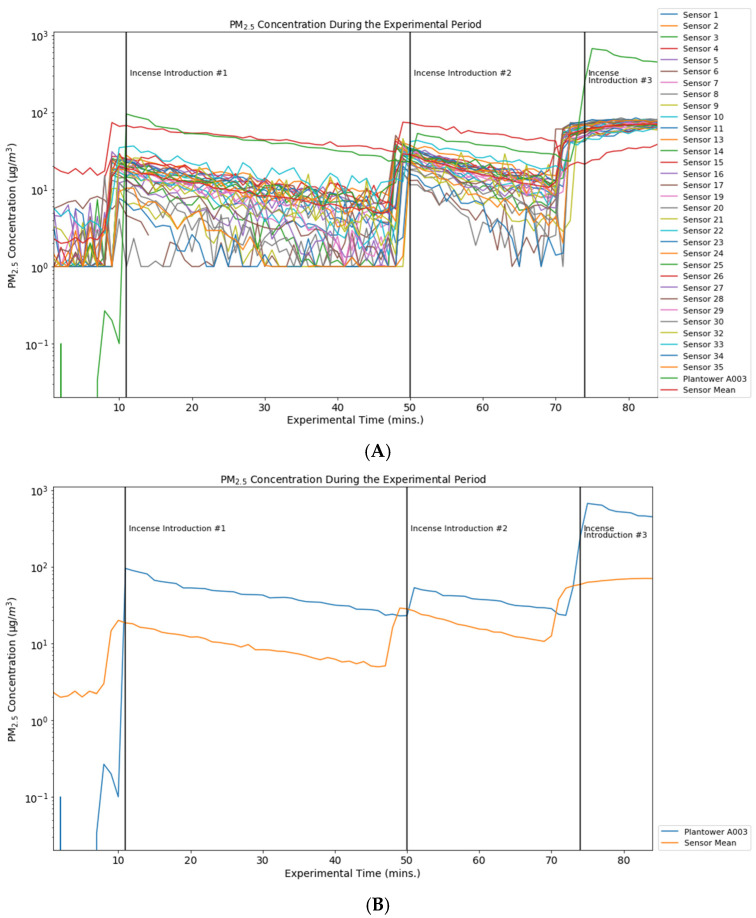
PM_2.5_ Concentration During the Experiemental Period Featuring Data from 32 Flow Sensors Individually (**A**) and in Aggregate (**B**) vs. Plantower A003.

**Figure 2:**
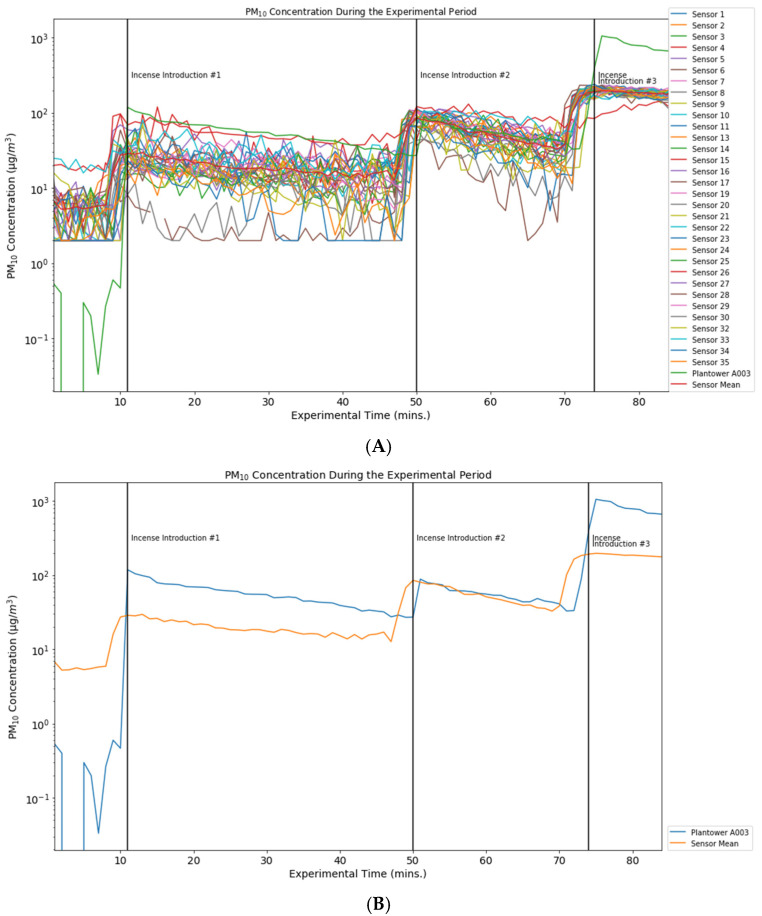
Caption: PM_10_ Concentration During the Experiemental Period Featuring Data from 32 Flow Sensors Individually (**A**) and in Aggregate (**B**) vs. Plantower A003.

PM_2.5_ and PM_10_ were generated in a controlled laboratory setting at Johns Hopkins University. In this setting, 34 charged and mobile device-paired Flow devices were co-located in an air-tight chamber (1.5 m × 1 m) with the Plantower A003 sensor. Our initial fleet of sensors was numbered 35 but one (Sensor 12) was excluded before the experiment because of a defect in the base of the sensor that made dock-charging infeasible. Incense was introduced into the chamber at 1:35 p.m. (experimental minute 11), 2:14 p.m. (experimental minute 50), and 2:38 p.m. (experimental minute 74) (Figure 1 and Figure 2). Data was collected from the Plantower A003 device every 30 s and output in Excel format. Flow devices collected PM_10_ and PM_2.5_ data every minute. Included in the observation period were the 10 min prior to the first introduction of incense to provide an initial PM-free baseline and 10 min after the last incense introduction. The chamber is provided with HEPA-filtered supply and exhaust air in order to be able to modify and test different concentrations. However, for the experiment described here, both supply and exhaust were turned off in order to increase the concentration and minimize dilution of the incense PM. 

### 2.3. Data Analysis

PM_10_ and PM_2.5_ data were downloaded from each of the 34 Flow devices in CSV format. The data from each of these sensors were matched to the nearest minute of data collection. Time was then converted from Universal Common Time (UTC) to Eastern Standard Time (EST). Our initial count of sensors was 35, but one broke before the experiment. Two of the Flow devices were found to be non-functional during the 84 min observational period, reducing the Flow sample size to 32. We did not alter the numbering scheme of our instruments for data management purposes. The Plantower A003, which collects data every 30 s, and the Flow average data, were matched to the nearest full minute. The mid-minute values were excluded from the analysis.

Analysis was performed using SAS 9.4 (SAS Enterprises, Cary, NC, USA), Python 3, and Stata/IC 13.1 (StataCorp, College Station, TX, USA).

### 2.4. Inter-Flow Variation

Two dimensions of variation were evaluated in the data collected for this experiment—among the devices over time and the devices in comparison with the Plantower A003 device. The standard deviation among the devices for each minute of the experiment was calculated and plotted. As in similar studies [6,18], the coefficient of variation (CV = $\frac{\sigma }{\mu }$) was evaluated for each minute of the experimental period to understand the variation among the 32 Flows. For PM_10_ and PM_2.5_, precision was assessed by calculating the relative precision error (RPE) between each possible pair of sensors that were functioning over the course of the experiment for each minute of the experiment (Equation (1)),

(1) $$\mathrm{RPE}=(|\frac{\mathrm{sensor}1-\mathrm{sensor}2}{\mathrm{average}\left(\mathrm{sensor}1,\;\mathrm{sensor}2\right)}|\;\times \;100)$$

RPE was then averaged for the duration of the experiment. These averaged paired values were then averaged by sensor to produce an overall RPE precision estimate for each sensor.

### 2.5. Flow and Plantower A003 Comparison

To understand how the average Flow measurements compared with that of the Plantower A003, the accuracy (Acc) of the Flows was evaluated by determining the absolute difference between the average Flow (sensor) and reference (Plantower A003) values, calculated using a method used by Levy Zamora et al. (2018) [18]. This value was then multiplied by 100 and then subtracted from 100 to generate an accuracy percentage value for each minute (Equation (2)). This value was then averaged to find the overall accuracy of each device. As some executions of the equation produced negative infinity values, these accuracy values were designated as non-numbers (NaNs) and processed as such.

(2) $$\mathrm{Acc}=(100-(|\frac{\mathrm{ref}.-\mathrm{sensor}}{\mathrm{ref}.}|\;\times \;100))$$

We then aggregated the accuracy across sensors for each minute to determine whether some periods of the experiment were more successfully measured than others. To evaluate the correlation between the Flow fleet and the Plantower A003, we produced a linear regression to examine the association between the Flows’ averaged minute-by-minute measurements and Plantower A003 datasets for each minute of the experimental period, yielding a Coefficient of Determination (R^2^) for the relationship between the Flows’ minute-by-minute average and that of the Plantower A003. We also produced scatter plots to visually assess correlation.

## 3. Results

Two devices, sensors 18 and 31, failed to measure changes in particulate matter. The minute-by-minute plots of the 32 remaining Flow devices and the Plantower A003 device, which has been well-studied, are presented for PM_2.5_ (Figure 1) and PM_10_ (Figure 2). 

**Figure 3:**
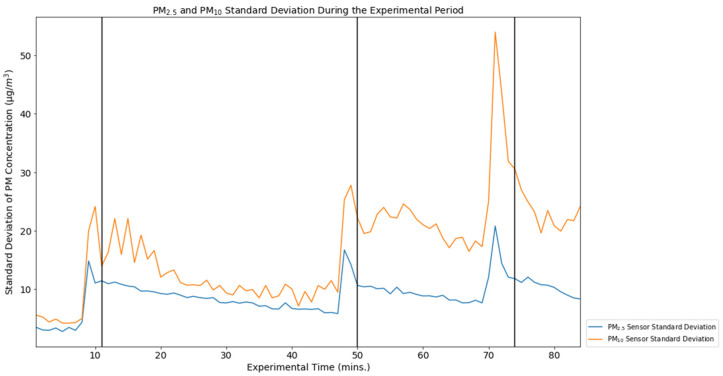
Flow PM_2.5_ and PM_10_ Average Standard Deviation During the Experimental Period.

These graphs present data for each of the sensors for the duration of the experiment, as well as an average of the Flow device measurements for each minute of the experiment. A separate graph displays the standard deviations of both the PM_10_ and PM_2.5_ measurements (Figure 3), which show the variability of the standard deviation by minute averaged across the 32 Flow devices. The Flows’ mean PM_2.5_ measurement over the course of the experiment was 20.15 µg/m^3^ (vs. Plantower measurement 99.51 µg/m^3^). The Flows’ mean PM_10_ measurement over the course of the experiment was 54.21 µg/m^3^ (vs. Plantower measurement 145.41 µg/m^3^).

The average CV values for PM_10_ and PM_2.5_ measurements among the Flow devices were 0.52 and 0.76, respectively. When compared to the Plantower A003, the overall accuracy of the Flow device’s PM_10_ measurements was −380.59% and that of the PM_2.5_ measurements was −433.72%. In the five minutes after each incense introduction, accuracy of PM_2.5_ measurements was higher, with averages of 21%, 47%, and 11% after incense introductions, 1, 2, and 3, respectively. In the five minutes after each incense introduction, accuracy of PM_10_ measurements was higher, with averages of 29%, 76%, and 21% after incense introductions, 1, 2, and 3, respectively. The overall RPE of each of the Flow devices’ measurements of PM_10_ and PM_2.5_ is presented in Table 1. 

The highest device RPE measured exceeded 100% and the lowest performing device had an overall precision in the 40% range for both PM_2.5_ and PM_10_ measurements. When measuring PM_2.5_, 22 devices (68.75%) had RPEs in the 40s–50s% range, 6 devices (18.75%) had RPEs in the 60s–70s% range and 4 devices (12.5%) had RPEs in the 80s–100s% range. When measuring PM_10_, 26 devices (81.25%) had RPEs in the 40s–50s% range, 3 devices (9.38%) had a precision in the 60s–70s% range and 3 devices (9.38%) had RPEs in the 80s–100s% range. The mean bias of the average Flow vs. Plantower A003 for PM_2.5_ measurements over the course of the experiment was 79.36 µg/m^3^. The mean bias of the average Flow vs. Plantower A003 for PM_10_ measurements over the course of the experiment was 91.20 µg/m^3^.

Regression analysis of the Plantower A003 and the average of the 32 Flows yielded a coefficient of determination (R^2^) of 0.73 for PM_10_ and a coefficient of determination (R^2^) of 0.76 for PM_2.5_. These results differ from those of South Coast AQMD [16], which used three Flow 2s that were analyzed individually as opposed to 32 Flows that were analyzed in aggregate.

## 4. Discussion

Analysis of the data of 32 Flow devices demonstrated differences in the measured concentrations of PM_10_ and PM_2.5_, both among the devices and in comparison with the Plantower A003 sensor. Among the Flow measurements, there was consistency in variation among devices in the context of PM_2.5_ measurement before and after incense introduction. The average standard deviation among sensors for PM_2.5_ and PM_10_ were 8.87 µg/m^3^ and 16.67 µg/m^3^, respectively (Table 2).

For PM_10_ measurements, however, this consistency was lacking, and became especially apparent when the incense was introduced to the test chamber during the experimental period. The CVs for PM_10_ and PM_2.5_ were 0.52 and 0.76, respectively, demonstrating a fair amount of variation in the measurement of each particulate matter fraction. Though each of the Flows records a measurement every minute, the specific second during which data are collected (and is time-stamped) varies. This is unlikely to be a problem for many of the measurements. However, if a Flow measurement were taken at the very beginning or end of a minute, then those reading may be more like those of either the preceding or following minute—variation among the Flows PM measurements may be due in part to this aspect of the Flow device’s function and may account for the peaks that appear in Figure 1, Figure 2 and Figure 3 before our recorded instances of incense introduction. 

In our evaluation of inter-device performance, we found variation in the overall precision (RPE) of each device in our fleet (Table 1). A few devices (4/32) performed with an overall PM_2.5_ concentration measurement precision of over 80%. The majority of the sensors, however, had an overall RPE in the 40s–50s% range over a PM_2.5_ concentration range of 1 to 300 µg/m^3^. Importantly, though there were different RPE measurements for each pollutant within each device, there was generally similar performance between PM_10_ and PM_2.5_, meaning a sensor that measured PM_10_ well also measured PM_2.5_ just as well. The likely explanation for this trend is that the same device within the sensor measures both PM_10_ and PM_2.5_. Understanding the overall RPE of devices is important when one deploys Flows for investigations that require device co-location; device co-location is often a necessary step in field studies when they are deployed to different locations to test and ensure adequate calibration and also to evaluate what part of the difference between sites is due to site concentration differences vs. due to sensor measuring difference [25,26].

We detected significant differences between the average measurement of the Flow devices and the Plantower A003 one minute after incense was introduced into the testing chamber (experimental minutes 12, 51, and 75). These differences, though substantial in some cases (Figure 1 and Figure 2), demonstrate a response to incense on the same timeline. In spite of the responsiveness of the sensors to particulate matter stimuli, there is relatively low accuracy when comparing the Flow average to the Plantower A003 sensor (Table 2).

In the immediate aftermath of the three incense introductions, average accuracy improved markedly (PM_2.5_: 21%, 47%, and 11%; PM_10_: 29%, 76%, and 21%). This poor total accuracy is largely attributable to the accuracy measurements that were calculated using Flow measurements that occurred during the first 10 min of the experimental period prior to incense introduction, which was demonstrated in high fluctuations in overall accuracy. The reason for the poor performance initially was the Flow’s measurement of particulate matter when clean filtered air was flowing through the chamber, which may be a product of the “learning” in which sensors engage as they are exposed to new environments. The overall accuracy of sensors dramatically improved with time and concentration increase compared with the initial assessment (i.e., Sensor 5 improved from −18,845% to 95% over the duration of the experimental period). In spite of this improvement, overall accuracy was relatively low; accuracy may be better assessed during known peak exposures, as demonstrated by the accuracy improvements in the five minutes after each of the incense introductions.

The inverted U-shaped trend produced in the PM_2.5_ and PM_10_ accuracy assessments during this period may suggest several different phenomena at work. The sensors may still be “learning” how to recognize high exposures well. The “learning” environments to which the sensors were exposed prior to this testing period were not extremely low or extremely high, consisting of generally low-to-moderate exposures. Some sensors may learn more quickly than others, which could also contribute to the variation in the measurements observed. Though there are substantial differences among Flows and between average Flow and Plantower A003 measurements, the Flows more effectively measured high particulate matter concentrations than at baseline, “clean” environments. Further, the sensors’ ability to measure changes in particulate matter concentration is reflected in the R^2^ and demonstrated visually through the PM_10_ and PM_2.5_ time series, which capture the overall temporal trends. 

The strengths in our approach include the number of Flow devices we were able to test and co-locate for this validation experiment, which made our analysis more robust. Further, we were granted access to a low-cost, high-precision sensor to evaluate the accuracy of the Flow devices. This equipment also allowed us to effectively test the Flow devices against each other to determine the RPE and accuracy of each device. 

Limitations of our approach include the short duration of this study (1.5 h), due largely to the amount of time needed to set up the Flow sensors, and the limited amount of instrument “burn-in” time that the Flows had, meaning that they were exposed to relatively few environments in a short period of time (i.e., indoor lab environment, indoor basement for a few days, car travel) vs. the recommended seven days [27] before this validation test began due to time and resource limitations. An experiment that assessed differences between largely “degreened” and not “degreened” sensors is needed to understand the effect of the self-learning in the measurement capacity of the sensors. This type of varied exposure is key to the device’s calibration, which relies on the device’s experience of ambient air environments [20]. This may have made it difficult for the Flow to recognize the lowest and highest concentrations of particulate matter that the Plantower A003 device was able to measure and the pronounced lack of concordance between the Flows and the Plantower A003 instrument during the first 10 min of the observational period. Extending and environmentally diversifying the “burn-in” period may improve the accuracy and precision of Flow readings across environments. The Flow devices’ pre-validation test environment was relatively consistent between the activation of the devices and the experiment.

As Castell et al. (2017) suggest regarding the utility of low-cost air quality sensors, Flows may have enhanced utility when their data is assessed in aggregate and in concentration when used as instruments in field campaigns [9]. Even if this mass deployment of sensors is not possible for an individual user, our analysis has demonstrated the Flow device’s ability to measure spikes in particulate matter pollution that are corroborated a previously evaluated robust sensor; being able to detect these spikes may be useful in protecting one’s respiratory health. Our analysis builds upon the preliminary evaluations already conducted [16] for the second generation of this sensor, which is presently being used in air quality, community health, and environmental justice research.

## 5. Conclusions

Flow sensors could be useful air quality monitoring tools in high-PM_2.5_ and -PM_10_ areas, such as cities, when incorporated into other monitoring frameworks, such as EPA monitoring networks, and when used in aggregate, with a number of sensors used as part of an air quality measurement campaign. The broader environmental implications of this work are that it is possible for individuals and groups to monitor their individual exposure to particulate matter pollution.

## Figures and Tables

**Table 1:** 31 comparisons were made among 32 sensors.

Overall Precision Evaluation	Overall Precision PM_10_	Overall Precision PM_2.5_
Sensor 1	47.61	54.13
Sensor 2	48.06	59.87
Sensor 3	45.66	47.36
Sensor 4	45.74	55.61
Sensor 5	48.53	50.53
Sensor 6	101.88	97.78
Sensor 7	45.44	49.03
Sensor 8	49.80	68.29
Sensor 9	60.25	79.81
Sensor 10	46.40	46.57
Sensor 11	47.63	47.41
Sensor 13	45.06	46.35
Sensor 14	50.49	59.08
Sensor 15	55.65	52.91
Sensor 16	56.10	51.72
Sensor 17	46.84	51.15
Sensor 19	48.44	50.73
Sensor 20	49.29	67.54
Sensor 21	49.73	50.29
Sensor 22	46.47	48.40
Sensor 23	49.68	50.33
Sensor 24	46.08	45.96
Sensor 25	45.89	49.65
Sensor 26	86.52	126.75
Sensor 27	45.96	60.82
Sensor 28	44.83	55.13
Sensor 29	47.59	48.61
Sensor 30	81.80	85.63
Sensor 32	54.47	53.87
Sensor 33	62.59	75.63
Sensor 34	70.46	87.40
Sensor 35	59.68	74.48

**Table 2:** Preliminary Analysis.

	PM_10_	PM_2.5_
Flow Average Standard Deviation	16.67 µg/m^3^	8.87 µg/m^3^
Flow Standard Deviation Minimum	4.20 µg/m^3^	2.77 µg/m^3^
Flow Standard Deviation Maximum	53.96 µg/m^3^	20.82 µg/m^3^
Flow Coefficient of Variation	0.52	0.76
Overall Accuracy (Flow Sensors vs. Plantower A003)	−380.59%	−433.72%
Linear Regression R^2^	0.73	0.76

## Data Availability

The data presented in this study are available on request from the corresponding author. The data are not publicly available due to their volume.
